# Effects of Elaidic Acid on HDL Cholesterol Uptake Capacity

**DOI:** 10.3390/nu13093112

**Published:** 2021-09-04

**Authors:** Takuya Iino, Ryuji Toh, Manabu Nagao, Masakazu Shinohara, Amane Harada, Katsuhiro Murakami, Yasuhiro Irino, Makoto Nishimori, Sachiko Yoshikawa, Yutaro Seto, Tatsuro Ishida, Ken-ichi Hirata

**Affiliations:** 1 Division of Cardiovascular Medicine, Graduate School of Medicine, Kobe University, Kobe 650-0017, Japan; iino@med.kobe-u.ac.jp (T.I.); mnishi.mail@gmail.com (M.N.); sachi.ys1228@gmail.com (S.Y.); y.seto0318@gmail.com (Y.S.); ishida@med.kobe-u.ac.jp (T.I.); hiratak@med.kobe-u.ac.jp (K.-i.H.); 2 Central Research Laboratories, Sysmex Corporation, 4-4-4 Takatsukadai, Nishi-ku, Kobe 651-2271, Japan; Harada.Amane@sysmex.co.jp (A.H.); Murakami.Katsuhiro@sysmex.co.jp (K.M.); Irino.Yasuhiro@sysmex.co.jp (Y.I.); 3 Division of Evidence-Based Laboratory Medicine, Graduate School of Medicine, Kobe University, Kobe 650-0017, Japan; mnagao@med.kobe-u.ac.jp; 4 Division of Epidemiology, Graduate School of Medicine, Kobe University, Kobe 650-0017, Japan; mashino@med.kobe-u.ac.jp; 5 The Integrated Center for Mass Spectrometry, Graduate School of Medicine, Kobe University, Kobe 650-0017, Japan

**Keywords:** high-density lipoprotein (HDL), cholesterol uptake capacity (CUC), phospholipids (PL), trans-fatty acids (TFA), elaidic acid, lecithin-cholesterol acyltransferase (LCAT)

## Abstract

Recently we established a cell-free assay to evaluate “cholesterol uptake capacity (CUC)” as a novel concept for high-density lipoprotein (HDL) functionality and demonstrated the feasibility of CUC for coronary risk stratification, although its regulatory mechanism remains unclear. HDL fluidity affects cholesterol efflux, and trans fatty acids (TFA) reduce lipid membrane fluidity when incorporated into phospholipids (PL). This study aimed to clarify the effect of TFA in HDL-PL on CUC. Serum was collected from 264 patients after coronary angiography or percutaneous coronary intervention to measure CUC and elaidic acid levels in HDL-PL, and in vitro analysis using reconstituted HDL (rHDL) was used to determine the HDL-PL mechanism affecting CUC. CUC was positively associated with HDL-PL levels but negatively associated with the proportion of elaidic acid in HDL-PL (elaidic acid in HDL-PL/HDL-PL ratio). Increased elaidic acid-phosphatidylcholine (PC) content in rHDL exhibited no change in particle size or CUC compared to rHDL containing oleic acid in PC. Recombinant human lecithin-cholesterol acyltransferase (LCAT) enhanced CUC, and LCAT-dependent enhancement of CUC and LCAT-dependent cholesterol esterification were suppressed in rHDL containing elaidic acid in PC. Therefore, CUC is affected by HDL-PL concentration, HDL-PL acyl group composition, and LCAT-dependent cholesterol esterification. Elaidic acid precipitated an inhibition of cholesterol uptake and maturation of HDL; therefore, modulation of HDL-PL acyl groups could improve CUC.

## 1. Introduction

High-density lipoprotein (HDL) is a multifunctional lipoprotein that protects against atherosclerosis. Although the detailed mechanisms are yet to be elucidated, a key function of HDL to protect cardiovascular events is suggested to be the efflux of cholesterol from macrophages in the arterial wall, which could be measured as cholesterol efflux capacity (CEC).

Previous studies have demonstrated a negative correlation between CEC and the probability of coronary artery disease (CAD) independent of HDL cholesterol (HDL-C) concentration [1,2,3]. However, since CEC assays require radiolabeled cholesterol and cultured cells and time consuming procedures [4,5], application of CEC in clinical settings is challenging. To overcome the technical limitations related to CEC, we recently established a simple, high-throughput, cell-free assay system to evaluate cholesterol uptake capacity (CUC) as a novel concept for HDL functionality. We have reported an inverse association between CUC and the recurrence rate of coronary lesions after revascularization in patients with optimal control of low-density lipoprotein cholesterol (LDL-C) concentrations [6,7]. However, the regulatory mechanism of CUC remains unclear.

Several studies have shown that the ability of HDL to accept cellular free cholesterol is related to the amount of phospholipids (PL) present in the particle [8,9], and that PL containing unsaturated fatty acids in their acyl groups increase the fluidity of the HDL surface and improve cholesterol efflux when incorporated into HDL [10]. In addition, we recently reported that oral administration of purified eicosapentaenoic acid (EPA) generates EPA-rich HDL particles, which exhibit cardioprotective properties via the production of anti-inflammatory lipid metabolites and an increase in cholesterol efflux [11,12]. These results indicate the importance of acyl groups of PL in HDL functionality.

Trans-fatty acids (TFA) are unsaturated fatty acids with at least one unsaturated double bond in the trans structure, whose excess intake is considered to be associated with an increased risk of cardiovascular disease (CVD) [13,14,15,16,17]. Previous studies have shown that TFA taken orally are incorporated into PL in plasma [18], where they reduce the fluidity of lipid membranes [19]. Considering that PL are the major lipid component of HDL [20], these results indicate the possibility that TFA are incorporated into the PL of HDL and affect its functionality. However, the relationship between CUC and TFA incorporated into HDL phospholipids (HDL-PL) has not yet been investigated. Therefore, the present study aimed to clarify the effect of TFA in HDL-PL on CUC.

## 2. Materials and Methods

### 2.1. Subjects

The Kobe Cardiovascular Marker Investigation (CMI) registry is a single-center registry of patients referred to Kobe University Hospital with cardiovascular disease, which is used to identify blood-based biomarkers that are useful in predicting cardiovascular disease. The study protocol was in accordance with the ethical guidelines of the 1975 Declaration of Helsinki. The study was approved by the Ethics Review Committee at Kobe University (Japan) and was registered in the UMIN Clinical Trials Registry (identification number 000030297). Written informed consent was obtained from all patients prior to enrollment in the study.

Serum samples were collected from patients who underwent coronary angiography (CAG) or percutaneous coronary intervention (PCI) and stored at 80 °C until measurement. The inclusion criteria for this study were patients with a history of PCI and follow-up CAG with or without revascularization between July 2015 and February 2019. Exclusion criteria were patients who did not have frozen serum samples for any reason.

### 2.2. Preparation of the apoB-Depleted Serum

Serum samples were thawed on ice and incubated with the same volume of 22% polyethylene glycol (PEG) 4000 to remove apolipoprotein B (apoB)-containing lipoproteins. Briefly, each serum sample was mixed with a PEG solution and kept at room temperature for 20 min. The samples were then centrifuged at 860× *g* for 15 min to precipitate all apoB-containing lipoproteins, and the supernatant was collected as apoB-depleted serum. A previous study that used gel filtration chromatography showed that cholesterol and PL colocalized in the same fraction as HDL in apoB-depleted serum [21]. Therefore, we used apoB-depleted serum for the HDL-PL analysis.

### 2.3. Clinical Variables

Serum levels of hemoglobin A1c (HbA1c), triglyceride (TG), total cholesterol (TC), LDL-C, HDL-C, and high-density lipoprotein triglyceride (HDL-TG) were measured using a standard assay at the Clinical Laboratory of Kobe University Hospital. HDL-PL levels were assessed by measuring apoB-depleted serum diluted eight times in phosphate-buffered saline (PBS) at SRL, Inc. (Hachioji, Tokyo, Japan) and calibrated using three-fold serially diluted pooled serum.

### 2.4. CUC Assay

The development of the CUC assay has been described previously [6,7]. In this study, the assay principle was applied to the HI-1000^TM^ system (Sysmex, Kobe, Japan), which is a fully automated immunoassay system for research applications. In brief, 5 μL of apoB-depleted serum was diluted in a buffer containing PBS and 0.2% R1 reagent of the HDL-C Reagent KL “kokusai” (Sysmex, Kobe, Japan) 200 times, and 10 μL of the diluted apoB-depleted serum was incubated with 90 μL of 1 μM biotin-PEG-labeled cholesterol (the preparation method is described in Appendix A) in reaction buffer (PBS containing 11% glycerol, 1.1% Pluronic F-68 (Thermo Fisher Scientific, Inc., Waltham, MA, USA), 0.11 mM methyl-β-cyclodextrin (Merck KGaA, Darmstadt, Germany), 0.055% liposome (Nippon fine chemical, Tokyo, Japan), 0.0047% nonion-K230 (NOF, Tokyo, Japan), 0.37% SF08 (NOF, Tokyo, Japan), and 0.009% oleamide (Kao, Tokyo, Japan)) at 37 °C for 1 min. Serum HDL was captured by an anti-apolipoprotein A1 (apoA1) mouse monoclonal antibody clone 8E10 (the preparation method is described in Appendix A) coated on magnetic particles at 37 °C for 6 min. After washing the particles with wash buffer (HISCL^TM^ line washing solution containing 0.1% Pluronic F-68 and 138 mM sodium chloride), 100 μL of alkaline phosphatase-conjugated streptavidin (Vector Laboratories, Burlingame, CA, USA) in dilution buffer (0.1 M TEA (pH 7.5) containing 10 mg/mL BSA, 5 mg/mL Casein Na, 1 mM MgCl_2_, and 0.1 mM ZnCl_2_) was added and incubated at 37 °C for 10 min. After washing the particles with wash buffer, the chemiluminescent substrate was added and incubated at 42 °C for 5 min, and chemiluminescence was measured as a count. The CUC assay was standardized using the pooled serum.

### 2.5. Measurement of Elaidic Acid Incorporated into HDL Phospholipids

One hundred microliters of 50 μM 1,2-dinonadecanoyl-sn-glycero-3-phosphocholine (19:0 PC; Merck KGaA, Darmstadt, Germany) were added to 200 μL of apoB-depleted serum as an internal standard, and total lipids were extracted using the Bligh and Dyer method as described previously [22] and applied to InertSep SI columns (GL Sciences Inc., Tokyo, Japan). The columns were then washed with 3 mL of chloroform and 3 mL of acetone. PL were eluted from the columns using 6 mL of methanol, dried under N_2_, and methylated with a commercially available kit (Nacalai Tesque, Kyoto, Japan) according to the manufacturer’s protocol. The concentrations of methylated elaidic acid were measured using gas chromatography-mass spectrometry (GC-MS). The GC-MS conditions used for the measurements in this study were described in a previous study [13], except that the split-less injection mode was adopted to increase the sensitivity, and each value was standardized using pooled serum.

### 2.6. Preparation of rHDL

The rHDL particles were prepared using a previously described sodium cholate dialysis method [12,23]. In brief, the required amounts of 1-palmitoyl-2-oleoyl-sn-glycero-3-phosphocholine (POPC) (Merck KGaA, Darmstadt, Germany), 1,2-dioleoyl-sn-glycero-3-phosphocholine (DOPC) (Merck KGaA, Darmstadt, Germany), or 1,2-dielaidoyl-sn-glycero-3-phosphocholine (elaidic acid PC) (Merck KGaA, Darmstadt, Germany), and cholesterol (FUJIFILM Wako Pure Chemical Corporation, Osaka, Japan) were mixed and dried under an N_2_ gas stream. The dried mixture was dissolved in tris(hydroxymethyl)aminomethane (Tris)-buffered saline (TBS; 8.2 mmol/L Tris-HCl, 150 mmol/L NaCl, pH 8.0) and supplemented with 19 mmol/L sodium deoxycholate until the solution was clear. ApoA1 from human plasma (Merck KGaA, Darmstadt, Germany) was added to the solution to make a final phosphatidylcholine (PC)–cholesterol–apoA1 molar ratio of 30:2:1. The mixture was incubated at 37 °C for 1 h and dialyzed against TBS for three days to remove sodium deoxycholate. The protein concentration was measured using the Bradford protein assay. The samples were subjected to non-denaturing 4–20% gradient polyacrylamide gel (Bio-Rad, Hercules, CA, USA) electrophoresis and stained with Coomassie Brilliant Blue to visualize the rHDL particles. Particle size was assigned by comparison with protein standards using a high molecular weight calibration kit (GE Healthcare, Madison, WI, USA).

### 2.7. CUC Assay for rHDL

rHDL was diluted in buffer to obtain a final apoA1 concentration of 1 μg/mL, and the CUC assay was performed with the HI-1000^TM^ system as described above. To evaluate the effects of lecithin-cholesterol acyltransferase (LCAT) on the CUC assay, recombinant human LCAT (rhLCAT) (Sino Biological Inc., Beijing, China) or rhLCAT preincubated with 2 mM N-ethylmaleimide (NEM) (FUJIFILM Wako Pure Chemical Corporation, Osaka, Japan) at 30 °C for 30 min were mixed with rHDL to make a final rhLCAT–apoA1 molar ratio of 1.5:1 or 4.2:1, respectively, and incubated at 37 °C for 5 min. The samples were then diluted in buffer to obtain a final apoA1 concentration of 1 μg/mL, and the CUC assay was performed. The quantification of apoA1 was conducted using the HI-1000^TM^ system and standardized using pooled serum. Briefly, an alkaline phosphatase-conjugated anti-apoA1 mouse monoclonal antibody clone P1A5 (the preparation method is described in Appendix A) was added to rHDL captured by an anti-apoA1 mouse monoclonal antibody (8E10)-coated on magnetic particles and incubated at 37 °C for 10 min. After washing the particles with wash buffer, the chemiluminescent substrate was added and incubated at 42 °C for 5 min, and chemiluminescence was measured as a count. To improve inter- and intra-assay precision, the CUC per apoA1 value was used for CUC analysis of rHDL.

### 2.8. Fluorescence-Based Assay for LCAT Activity

A fluorescence-based assay for LCAT activity was developed according to a previous study [24]. The rHDL particles containing POPC or elaidic acid-PC, BODIPY-cholesterol (Avanti Polar Lipids, Alabaster, AL, USA), and apoA1 in a ratio of 30:2:1 were prepared and used as proteoliposome substrates. The samples were subjected to non-denaturing 4–20% gradient polyacrylamide gel (Bio-Rad, Hercules, CA, USA) electrophoresis and analyzed with a ChemiDoc Touch MP (Bio-Rad, Hercules, CA, USA) set at 488 nm for excitation and 520 nm for emission to detect BODIPY-cholesterol. The same gel was stained with Coomassie Brilliant Blue to visualize the rHDL particles.

The rhLCAT or rhLCAT preincubated with 2 mM NEM at 30 °C for 30 min was mixed with the proteoliposome substrates to make a final rhLCAT:apoA1 molar ratio of 0.5:1, and incubated at 37 °C for 10–90 min. The lipids were extracted from the samples, dissolved in 30 µL of chloroform, and applied to a thin-layer chromatography (TLC) silica gel 60 plate (Merck KGaA, Darmstadt, Germany), which was then placed into a closed glass tank and saturated with a developing solvent (petroleum ether, diethyl ether, and acetic acid in mole portions of 230:60:3). After 25 min, the TLC plate was removed from the tank and cholesterol spots and esterified cholesterol spots were detected using a ChemiDoc Touch MP set at 488 nm for excitation and 520 nm for emission. For quantitative analysis of cholesterol esterification rate, the TLC plate was exposed for 0.2 s and the fluorescence intensities of both cholesterol spots and esterified cholesterol spots were quantified by densitometry analysis using ImageJ^®^ software (NIH, Bethesda, MD, USA). The cholesterol esterification rate was calculated using the following formula:

% Cholesterol esterification rate = (Fluorescence intensities of esterified BODIPY-cholesterol spots derived from each rHDL/Fluorescence intensities of BODIPY-cholesterol spots derived from rHDL without addition of rhLCAT) × 100.

For visual inspection, exposure time for detecting BODIPY-cholesterol and esterified BODIPY-cholesterol spots were set to 0.2 and 3.0 s, respectively.

### 2.9. Statistics

Statistical analyses of clinical subjects were performed using Stata 16.1 (StataCorp LLC, College Station, TX, USA), and for rHDL, the GraphPad Prism software version 8.4.3 (GraphPad Software, Inc., San Diego, CA, USA). Categorical variables were expressed as numbers and percentages, and the *p* value for differences between two groups was determined using the Chi-square test. Continuous variables were expressed as mean ± standard deviation (SD), unless otherwise specified. The *p* value for differences between two groups was determined by an unpaired Student’s t-test or the Mann–Whitney test according to the data distribution and normality. Differences between multiple groups were determined by one-way ANOVA with Tukey’s or Dunnett’s multiple comparisons test, as applicable. The relationships between the two numerical variables were investigated using a simple linear regression analysis. We report Spearman’s rho with corresponding *p* values. Statistical significance was set at *p* < 0.05.

## 3. Results

### 3.1. Baseline Patient Characteristics

From the Kobe CMI registry between July 2015 and February 2019, we enrolled 264 patients based on the inclusion and exclusion criteria. The baseline patient characteristics and laboratory data are shown in Table 1.

More than 80% of the patients were receiving statin therapy, and achieved a mean LDL-C level of less than 100 mg/dL, which is the goal for secondary prevention of coronary artery disease (CAD) in Japan [25]. The patients in the revascularization (Rev.(+) group had a significantly higher incidence of diabetes than patients without revascularization (Rev.(–)). Conversely, CUC and HDL-PL levels were significantly higher in the Rev.(–) patients than those in the Rev.(+) group. Elaidic acid levels in HDL-PL also tended to be higher in the Rev.(–) group than in the Rev.(+) group, although this trend was not statistically significant (Supplemental Tables S1 and S2).

### 3.2. The Proportion of Elaidic Acid in HDL-PL Inversely Correlates with CUC

**Figure 1:**
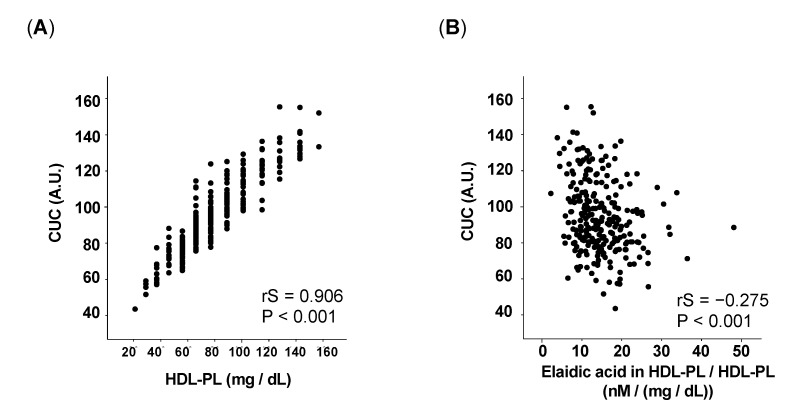
Correlations between CUC and the following: (**A**) HDL-PL levels (rS = 0.906, *p* < 0.001), (**B**) elaidic acid in HDL PL/HDL-PL ratio (rS = −0.275, *p* < 0.001). CUC, cholesterol uptake capacity; A.U., arbitrary units; HDL-PL, high-density lipoprotein phospholipid.

As a first step towards understanding the effect of TFA in HDL-PL on CUC, we assessed the relationship between CUC and HDL-PL and confirmed that CUC was positively associated with HDL-PL levels (rS = 0.906, *p* < 0.001) (Figure 1A). Though CUC was also positively associated with apoA1 levels (rS = 0.683, *p* < 0.001) (Figure S1A), the value of correlation coefficient was smaller than that of HDL-PL levels, suggesting that the HDL-PL level is an important factor in determining CUC.

To analyze the effect of TFA incorporated into HDL-PL on CUC, we evaluated the relationship between CUC and elaidic acid in HDL-PL and found that although there was a positive correlation (Figure S1B); CUC was negatively associated with the proportion of elaidic acid in the HDL-PL/HDL-PL ratio (rS = −0.275, *p* < 0.001) (Figure 1B). By contrast, though oleic acid, a cis analogue of elaidic acid, in HDL-PL also correlated positively with CUC (Figure S1C), no significant relationship was noted between CUC and the proportion of oleic acid in HDL-PL (Figure S1D). These results indicate the possibility that elaidic acid has a negative effect on CUC when incorporated into HDL-PL.

### 3.3. LCAT-Dependent Enhancement of CUC Is Suppressed in rHDL Containing Elaidic Acid-PC

**Figure 2:**
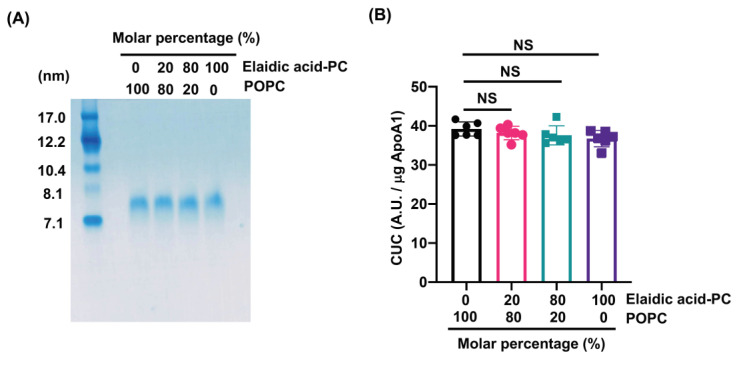
Elaidic acid-PC contents in rHDL do not affect particle size and CUC. (**A**) rHDL containing different POPC and elaidic acid-PC molar percentages was prepared and native polyacrylamide gel electrophoresis (PAGE) analysis was performed in a 4–20% polyacrylamide gel to assess the particle size. Standard proteins of known hydrodynamic diameters were used for this analysis. Samples (1.0 μg proteins) were separated by non-denaturing gel electrophoresis and stained with Coomassie Brilliant Blue. (**B**) rHDL containing different POPC and elaidic acid-PC molar percentages was prepared and CUC assay was performed. Values are expressed as the mean ± SD (*n* = 6). CUC, cholesterol uptake capacity; A.U., arbitrary units; NS, not significant. Data analyzed by one-way ANOVA with Dunnett’s multiple comparisons test.

To investigate the effects of elaidic acid in HDL-PL on HDL size and functionality, discoidal rHDL containing various molar percentages of POPC and elaidic acid-PC (0–100% of total PC) were prepared and particle size and CUC were assessed. Native PAGE analysis showed that particle sizes did not differ significantly between rHDLs (Figure 2A).

Similarly, contrary to our expectation, the elaidic acid-PC content in rHDL did not affect CUC (Figure 2B), although these results might have been due to the limitations of CUC analysis using only rHDL.

Under physiological conditions, LCAT is known to bind discoidal small HDLs (pre-β-HDL) [26,27] and is important for HDL maturation [28]. In peripheral tissues, free cholesterol effluxes from cells by the ATP-binding cassette transporter A1 (ABCA1) to pre-β-HDL and is esterified by LCAT. Due to their hydrophobic chemical properties, cholesterol esters (CE) move to the core of the HDL [29], making it larger and more spherical mature. Recently, it has been reported that rhLCAT increased CE and enhanced cholesterol efflux and the maturation of HDL in vivo [30]. Therefore, we hypothesized that the addition of rhLCAT to rHDL would enable CUC analysis under near-physiological conditions.

**Figure 3:**
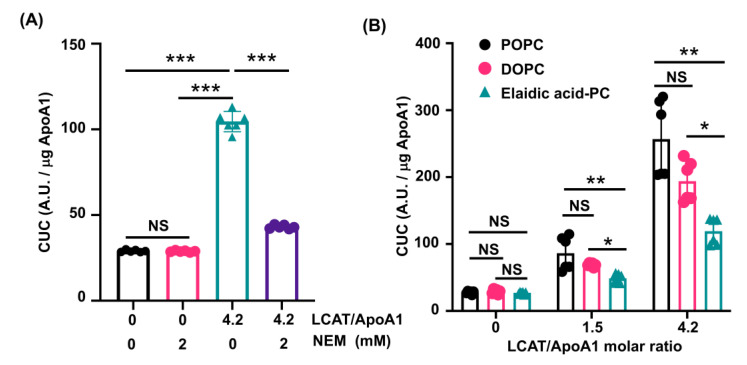
LCAT-dependent enhancement of CUC is suppressed in rHDL containing elaidic acid-PC. (**A**) rHDL containing POPC was prepared and a CUC assay was performed in the presence of rhLCAT or rhLCAT pre-incubated with NEM. Values are expressed as the mean ± SD (*n* = 6). LCAT, lecithin cholesterol acyltransferase; apoA1, apolipoprotein A1; NEM, N-ethylmaleimide; CUC, cholesterol uptake capacity; A.U., arbitrary units. *** *p* < 0.001. NS, not significant. Data analyzed by one-way ANOVA with Tukey’s multiple comparisons test. (**B**) rHDL containing POPC, DOPC, and elaidic acid-PC was prepared and a CUC assay was performed in the presence of rhLCAT. Values are expressed as the mean ± SD (*n* = 6). * *p* < 0.05, ** *p* < 0.01. NS, not significant. Data analyzed by one-way ANOVA with Dunnett’s multiple comparisons test.

To investigate the effects of LCAT on CUC, rHDL containing POPC was prepared and the CUC assay was performed in the presence of rhLCAT or rhLCAT pre-incubated with NEM, which inhibits LCAT activity [31,32,33,34]. The addition of rhLCAT to rHDL significantly enhanced CUC, and LCAT-dependent enhancement of CUC was suppressed by NEM (Figure 3A).

Next, to investigate the effects of elaidic acid in HDL-PL on CUC in the presence of LCAT, rHDL containing POPC, DOPC, and elaidic acid-PC were prepared and the CUC assay was performed in the presence of rhLCAT. Although PL contain saturated fatty acids mainly in the sn1 position [35,36,37], we used a PC containing elaidic acid in both the sn1 and sn2 positions as elaidic acid-PC. To confirm the effect of sn1 substitution by monounsaturated fatty acids, DOPC, which contains oleic acid in both the sn1 and sn2 positions, and POPC, which contains palmitic acid in the sn1 position and oleic acid in the sn2 position, were used as controls. Although rhLCAT-dependent enhancement of CUC was observed in all rHDLs, the CUC of rHDL containing elaidic acid-PC was significantly lower than that of rHDL containing POPC or DOPC (Figure 3B). These findings indicate that LCAT plays a crucial role in the enhancement of CUC, and elaidic acid has a negative effect on CUC in the presence of LCAT.

### 3.4. LCAT-Dependent Cholesterol Esterification Is Suppressed in rHDL Containing Elaidic Acid-PC

**Figure 4:**
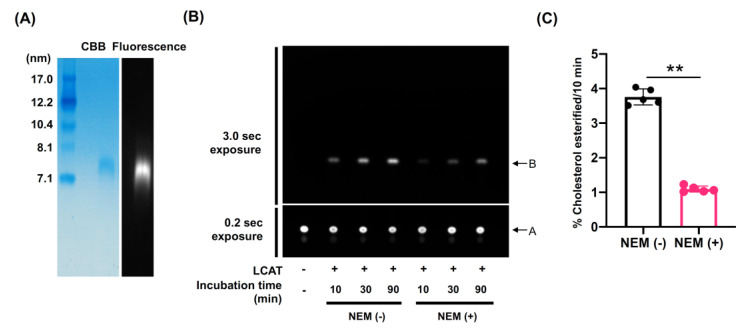
Development of a fluorescence-based assay for LCAT activity. (**A**) rHDL containing both BODIPY-cholesterol and POPC was prepared and native PAGE analysis was performed in a 4–20% polyacrylamide gel. Standard proteins of known hydrodynamic diameters were used for the analysis. Samples (1.0 μg proteins) were separated by non-denaturing gel electrophoresis and stained with Coomassie Brilliant Blue (left). The same Native PAGE gel was analyzed with a ChemiDoc Touch MP (Bio-Rad) set at 488 nm for excitation and 520 nm for emission (right). (**B**) rhLCAT or rhLCAT pre-incubated with NEM was incubated with rHDL containing BODIPY-cholesterol and POPC for 10–90 min at 37 °C. The extracted lipids were dissolved in 30 µL of chloroform and applied to the TLC plate. The TLC plate was placed into a closed glass tank, saturated by a developing solvent (petroleum ether, diethyl ether, and acetic acid in mole portions of 230:60:3). After 25 min, the plate was removed and the cholesterol spots (Position A) and esterified cholesterol spots (Position B) were detected using a ChemiDoc Touch MP set at 488 nm for excitation and 520 nm for emission. In order to visualize spots clearly, cholesterol spots were exposed for 0.2 s and esterified cholesterol spots were exposed for 3.0 s. (**C**) The TLC plate was exposed for 0.2 s and cholesterol spots and esterified cholesterol spots were quantified by densitometry analysis using ImageJ^®^ software. Cholesterol esterification rate was calculated as the percentage of cholesterol esterified during HDL incubation at 37 °C in 10 min. Values are expressed as the mean ± SD (*n* = 5). ** *p* < 0.01. Data analyzed by unpaired Mann–Whitney test.

Previous studies have shown that conversion of free cholesterol on HDL to CE by LCAT increases the capacity of HDL to remove additional cholesterol and maintains the gradient for cholesterol efflux from cells [29,30]. Therefore, we speculated that elaidic acid in HDL-PL inhibited LCAT-dependent cholesterol esterification on HDL and affected CUC. To evaluate LCAT-dependent cholesterol esterification, a fluorescence-based assay for LCAT activity was developed according to a previous study [24]. First, we prepared rHDL containing both BODIPY-cholesterol and POPC as a proteoliposome substrate and confirmed that the fluorescent signal was detected in the same size as rHDL by native PAGE analysis (Figure 4A).

Second, rhLCAT or rhLCAT pre-incubated with NEM was incubated with rHDL for 10–90 min, both BODIPY-cholesterol and esterified BODIPY-cholesterol were separated by TLC, and fluorescent signals were detected. Fluorescent intensities of esterified BODIPY-cholesterol increased depending on the incubation time in the presence of rhLCAT, and this trend was suppressed by pre-incubation of rhLCAT with NEM (Figure 4B). Quantitative analysis also showed that the LCAT-dependent cholesterol esterification rate was suppressed by NEM (Figure 4C). We concluded from these results that the fluorescence activity assay for LCAT developed properly.

**Figure 5:**
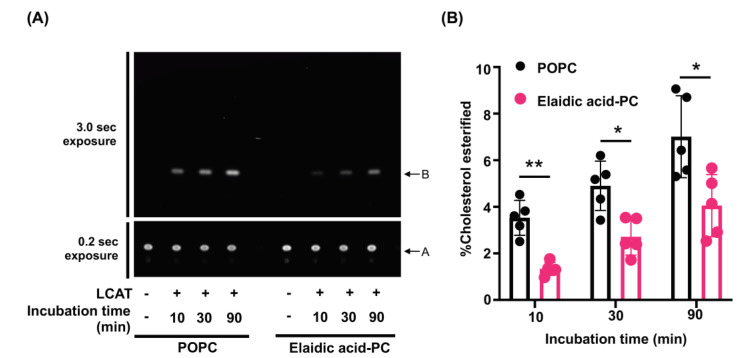
LCAT-dependent cholesterol esterification is suppressed in rHDL containing elaidic acid-PC. (**A**) rHDL containing both BODIPY-cholesterol and POPC or elaidic acid-PC were prepared and incubated with rhLCAT for 10–90 min at 37 °C. The extracted lipids were dissolved in 30 µL of chloroform and applied to the TLC plate. The TLC plate was placed into a closed glass tank, saturated by a developing solvent (petroleum ether, diethyl ether, and acetic acid in mole portions of 230:60:3). After 25 min, the plate was removed and the cholesterol spots (Position A) and esterified cholesterol spots (Position B) were detected using a ChemiDoc Touch MP. In order to visualize the spots clearly, cholesterol spots were exposed for 0.2 sec and esterified cholesterol spots were exposed for 3.0 sec. (**B**) The TLC plate was exposed for 0.2 sec and cholesterol spots and esterified cholesterol spots were quantified by densitometry analysis using ImageJ^®^ software. Cholesterol esterification rate was calculated as the percentage of cholesterol esterified during HDL incubation at 37 °C for, 10–90 min. Values are expressed as the mean ± SD (*n* = 5). * *p* < 0.05 ** *p* < 0.01. Data analyzed by unpaired Mann–Whitney test.

Finally, to evaluate the effect of elaidic acid in HDL-PL on LCAT-dependent cholesterol esterification, rHDL containing both BODIPY-cholesterol and POPC or elaidic acid-PC were prepared as proteoliposome substrates and a fluorescence activity assay for LCAT was performed. Although the fluorescent intensities of esterified BODIPY-cholesterol increased depending on the incubation time in the presence of LCAT in both rHDLs (Figure 5A), the cholesterol esterification rate of rHDL containing elaidic acid-PC was significantly lower than that of rHDL containing POPC (Figure 5B), demonstrating that elaidic acid suppresses esterification of cholesterol on HDL when incorporated into HDL-PL.

## 4. Discussion

In this study, we demonstrated that CUC, a novel indicator of HDL functionality, was inversely associated with the proportion of elaidic acid in HDL-PL. In vitro analysis using rHDL showed that rhLCAT enhanced CUC, and LCAT-dependent enhancement of CUC was suppressed in rHDL containing elaidic acid in PC compared to rHDL containing oleic acid, a cis analogue of elaidic acid. Moreover, we found that LCAT-dependent cholesterol esterification was also suppressed by elaidic acid.

PL are major components of the HDL lipidome, accounting for 40–60% of total HDL lipids, followed by cholesteryl esters (30–40%), triglycerides (5–12%), and free cholesterol (5–10%) [20]. In the present study, we found that HDL-PL levels were strongly significantly correlated with CUC, which agrees with previous studies that showed a significantly positive correlation between CEC and HDL-PL levels [38]. In vitro analysis using rHDL also showed that CEC at a fixed rHDL protein concentration increased in parallel with increasingly enriched PL [39]. Since cholesterol interacts with PL [40], the latter are indispensable components for maintaining cholesterol in lipid membranes. We believe that our results reflect the intrinsic mechanism of the affinity between PL and cholesterol.

Previously, we showed that serum elaidic acid levels were elevated in middle-aged patients with CAD and/or metabolic syndrome in Japan [13]. We also showed that elevated serum elaidic acid levels were associated with the incidence of target vessel revascularization (TLR) in the same-age Japanese generation with CAD [14]. Dietary TFA are reported to be associated with increased LDL-C and TG, as well as reduced HDL-C [41], suggesting that the adverse effects of TFA on lipoprotein quantity and function may contribute to the increase in CVD events. Nevertheless, the effects of TFA on HDL functionality have not been completely elucidated.

In this study, both CUC and HDL-PL levels were significantly higher in the Rev.(–) group than in the Rev.(+) group. Accompanied by the increase in HDL-PL levels, the elaidic acid levels in HDL-PL also tended to be higher in the Rev.(–) group than in the Rev.(+) group. However, this trend was not statistically significant. To investigate the effect of the elaidic acid composition of HDL-PL on CUC, we examined the relationship between the proportion of elaidic acid in HDL-PL (elaidic acid in HDL-PL/HDL-PL ratio) and CUC, and found a negative correlation. By contrast, oleic acid, a cis analogue of elaidic acid, showed no such relationship. These results suggest that not only the amount of PL but also the composition of PL is a factor in determining CUC, and that the increased proportion of elaidic acid in HDL-PL has a negative effect on CUC.

In the present study, we found that the addition of rhLCAT to rHDL enhanced CUC, and LCAT-dependent enhancement of CUC was suppressed in rHDL containing elaidic acid in PC when compared to rHDL containing oleic acid, a cis analogue of elaidic acid. A previous study showed that the incorporation of structurally linear elaidic acid into PL reduces the fluidity of lipid membranes [19]. Therefore, elaidic acid could reduce the surface fluidity of HDL and attenuate CUC in the presence of LCAT. Additionally, the present study showed that LCAT was less reactive to PC containing elaidic acid than PC containing oleic acid, and affected the efficiency of cholesterol esterification in rHDL. A previous study showed that cholesterol esterification contributed to HDL maturation and increased the capacity of HDL to remove cholesterol [29,30]. Therefore, elaidic acid may affect the esterification of cholesterol in addition to membrane fluidity, thereby inhibiting cholesterol uptake and maturation of HDL. Although the mechanism by which elaidic acid affects LCAT reactivity has not been fully elucidated, considering that substrates of PL need to move into the active site of LCAT from HDL through the path that is made by the interaction between LCAT and apoA1 [42], elaidic acid may decrease the surface fluidity of HDL and reduce the efficiency of providing substrates of PL to LCAT through the path.

In this study, we did not perform a detailed structural analysis to elucidate how rHDL, which contains elaidic acid-PC, undergoes structural changes upon reaction with rhLCAT. Recently, the binding mode of LCAT and HDL was analyzed using negative stain electron microscopy (EM), validated with hydrogen–deuterium exchange mass spectrometry (HDX-MS) and crosslinking coupled with mass spectrometry (XL-MS) [42]. Adaptation of these techniques for rHDL analysis may reveal more detailed effects of elaidic acid-PC on LCAT-dependent HDL maturation in the future.

Recently, much attention has been focused on restoring or regulating HDL function to prevent atherosclerosis. Previously, we found that EPA enhanced CEC when it was incorporated into HDL [11,12]. In the present study, we found that elaidic acid incorporated in HDL-PL negatively affected CUC. In view of these results, modulation of the PL acyl groups may be an effective strategy to improve HDL function. CUC was significantly enhanced in the presence of rhLCAT. Recently, therapeutic concepts for coronary heart disease and atherosclerosis using recombinant LCAT protein or an LCAT activator have been proposed, and dose-dependent increases in HDL-C along with the enhancement of cholesterol efflux or in vivo reverse cholesterol transport (RCT) have been demonstrated [30,43]. Considering the enhancement of CUC in the presence of rhLCAT, as shown in this study, CUC may change in response to these molecules, in a manner similar to cholesterol efflux.

### Study Limitations

This study has several limitations. First, because CUC is determined by a cell-free assay, CUC does not reflect the ABCA1 mediated cellular binding of apoA1 and the unidirectional export of cholesterol and PL to lipid-free/-poor apoA1 [7], which is considered as the first step of reverse cholesterol transport. Hence, the effect of elaidic acid in HDL-PL on cholesterol efflux remains to be elucidated. Second, we assessed rHDL containing only elaidic acid in PC for in vitro analysis. Since the concentrations of elaidic acid in vivo are much lower than those of other fatty acids, HDL containing such a highly enriched elaidic acid does not exist in vivo. However, considering the inverse association between CUC and the proportion of elaidic acid in HDL-PL observed in the correlation study using serum samples, we believe that our results reflect the intrinsic effect of elaidic acid on HDL. Further elucidation is required to address this issue. Third, although we used PC, which contains elaidic acid in both the sn1 and sn2 positions, as elaidic acid-PC, it is not consistent with a previous study that showed that PL contains saturated FA at position sn1 and unsaturated FA at position sn2 [35,36,37]. However, a previous study that assessed the membrane fluidity by steady-state fluorescence polarization of the probe diphenylhexatriene (DPH) showed that lipid membranes made from trans-containing PC (trans-PC) were less fluid than lipid membranes made from cis-containing PC (cis-PC), regardless of the position of incorporation (sn1 only, or both sn1 and sn2 of the glycerol backbone) [19]. Hence, we believe that the type of elaidic acid-PC used in our rHDL analysis did not affect the conclusions of this study. Fourth, we assessed rHDL containing the same amount of apoA1 for in vitro analysis. Since the interaction of LCAT to apoA1 enhances the enzymatic activity of LCAT [42], the amount of apoA1 per HDL particle and post-translational modifications of apoA1 such as nitration [44] may affect the LCAT-dependent cholesterol esterification and CUC. To address this issue, a comprehensive analysis using rHDL containing different amounts and qualities of apoA1 is needed.

Lastly, we did not assess the effects of polyunsaturated fatty acids, which may enhance lipid membrane fluidity. Further studies, such as comprehensive lipid profile assessment of HDL and analysis of rHDL composed of other types of phospholipids are needed to generalize the present findings.

## 5. Conclusions

The present study revealed that CUC is affected by the HDL-PL level. Moreover, CUC was negatively associated with the proportion of elaidic acid in HDL-PL, suggesting that the composition of HDL-PL is also a determinant factor of CUC. In vitro analysis using rHDL showed that CUC was positively affected by LCAT-dependent cholesterol esterification, whereas the incorporation of elaidic acid in HDL-PL attenuated the cholesterol esterification efficiency by LCAT in addition to decreasing the fluidity of the HDL surface as reported previously, thereby inhibiting the process of cholesterol uptake and maturation of HDL. Further analysis to elucidate the regulatory mechanisms of CUC will lead to new diagnostic and therapeutic strategies for atherosclerosis and cardiovascular disease.

## Figures and Tables

**Table 1:** Baseline patient characteristics and laboratory data.

Variables	*n* = 264
Age	70.8 ± 9.3
Male, *n* (%)	210 (79.5)
Hypertension, *n* (%)	204 (77.3)
Dyslipidemia, *n* (%)	221 (83.7)
Diabetes, *n* (%)	119 (45.1)
Smoking history, *n* (%)	180 (68.4)
Statin, *n* (%)	233 (88.2)
Laboratory data	
HbA1c (%)	6.4 ± 1.0
TG (mg/dL)	128.8 ± 71.2
TC (mg/dL)	146.8 ± 31.2
LDL-C (mg/dL)	82.1 ± 26.3
HDL-C (mg/dL)	46.1 ± 12.6
CUC (A.U.)	94.8 ± 20.5
ApoA1 (mg/dL)	118.0 ± 19.3
HDL-PL (mg/dL)	78.0 ± 26.6
HDL-TG (mg/dL)	13.6 ± 6.6
Elaidic acid in HDL-PL (μM)	1.1 ± 0.50

Values are presented as mean ± SD. HbA1c, hemoglobin A1c; TG, triglyceride; TC, total cholesterol; LDL-C, low-density lipoprotein cholesterol; HDL-C, high-density lipoprotein cholesterol; CUC, cholesterol uptake capacity; ApoA1, apolipoprotein A1; HDL-PL, high-density lipoprotein phospholipid; HDL-TG, high-density lipoprotein triglyceride; A.U., arbitrary units.

## Data Availability

Not applicable.

## Supplementary Materials

The following are available online at https://www.mdpi.com/article/10.3390/nu13093112/s1, Figure S1: Correlations between CUC and elaidic acid or oleic acid in HDL-PL; Table S1: Detailed laboratory data; Table S2: Detailed baseline patient characteristics.

## Appendix A. Supplemental Methods

### Appendix A.1. Generation of Mouse Monoclonal Antibody 8E10 and P1A5

Hybridoma cell lines were generated by immunizing C57BL/6 mice with recombinant human apoA1 protein (Merck KGaA, Darmstadt, Germany). Mouse immunization and generation of hybridoma cell lines were outsourced to the Cell Engineering Corporation (Osaka, Japan). Hybridoma culture supernatants containing antibodies with the desired binding specificity for equal recognition of non-oxidized and oxidized HDL were screened by ELISA. In brief, 1 μg/mL of recombinant human apoA1 protein or apoB-depleted serum with an apoA1 concentration of 1 μg/mL, diluted in PBS, were immobilized on 96-well plates at 37 °C for 1 h. After washing the wells with PBS, PBS with or without hydrogen peroxide (H_2_O_2_), sodium nitrite, and diethylenetriaminepentaacetic acid (DTPA) solution (final concentrations of 1 mol/L, 200 μmol/L, and 100 μmol/L, respectively) were added to the wells and incubated at 37 °C for 1 h. The wells were washed with PBS and blocked with 2% BSA in PBS at 25 °C for 1 h. The plates were then incubated with hybridoma culture supernatant at 25 °C for 1 h, followed by the addition of horseradish peroxidase (HRP)-conjugated goat anti-mouse IgG (Dako, Glostrup, Denmark) at 25 °C for 30 min. The wells were washed with PBS five times, SuperSignal ELISA pico chemiluminescent substrate (Thermo Fisher Scientific, Inc., Waltham, MA, USA) was added to the wells, and the chemiluminescence signal was measured using an Infinite F200 Pro microplate reader (Tecan, Mannedorf, Switzerland). The mAb 8E10 and P1A5 were selected by screening for equal recognition of lipid-free (recombinant protein) and lipidated (apoB-depleted serum) apoA1 under native conditions, as well as after oxidation by exposure to H_2_O_2_/NO_2_^−^. In order to obtain sufficient antibodies for this study, mAb 8E10 was purified from the ascites fluid of ICR nude mice by Protein A-Sepharose chromatography. Preparation of mouse ascites fluid and purification of mAb 8E10 and P1A5 were outsourced to Kitayama Labes (Nagano, Japan).

### Appendix A.2. Synthesis of Biotin-PEG7-Cholesterol

Fifteen milligrams of 3β-Hydroxy-Δ^5^-cholenic Acid (Wako) were dissolved in 500 μL of N,N-dimethylformamide. Then, 7.7 mg of 1-Ethyl-3-(3-dimethylaminopropyl)carbodiimide, hydrochloride (Dojindo), 4.6 mg of N-hydroxysuccinimide (Merck KGaA, Darmstadt, Germany), 23.8 mg of Biotin-PEG7-amine (BroadPharm), and 8.4 μL of triethylamine (Wako) were added, and the resulting solution was stirred at room temperature for 2 h. Silica gel column chromatography (10% methanol in chloroform) yielded Biotin-PEG7-cholesterol as a clear solid (4% yield). LC-MS (*m/z*): 951.4 [M + H]^+^.
