# Supplementary material for: Effects of Elaidic Acid on HDL Cholesterol Uptake Capacity
Source: Nutrients. 2021 Sep 4;13(9):3112. doi: 10.3390/nu13093112 (PMC8464738; doi:10.3390/nu13093112)
Supplement: Supplementary file 1 [file nutrients-13-03112-s001.zip › nutrients-1336097-supplementary/Supplemetary files (final prrof)/Nutrients-1336097_Supplemental Figures (final proof).pdf]

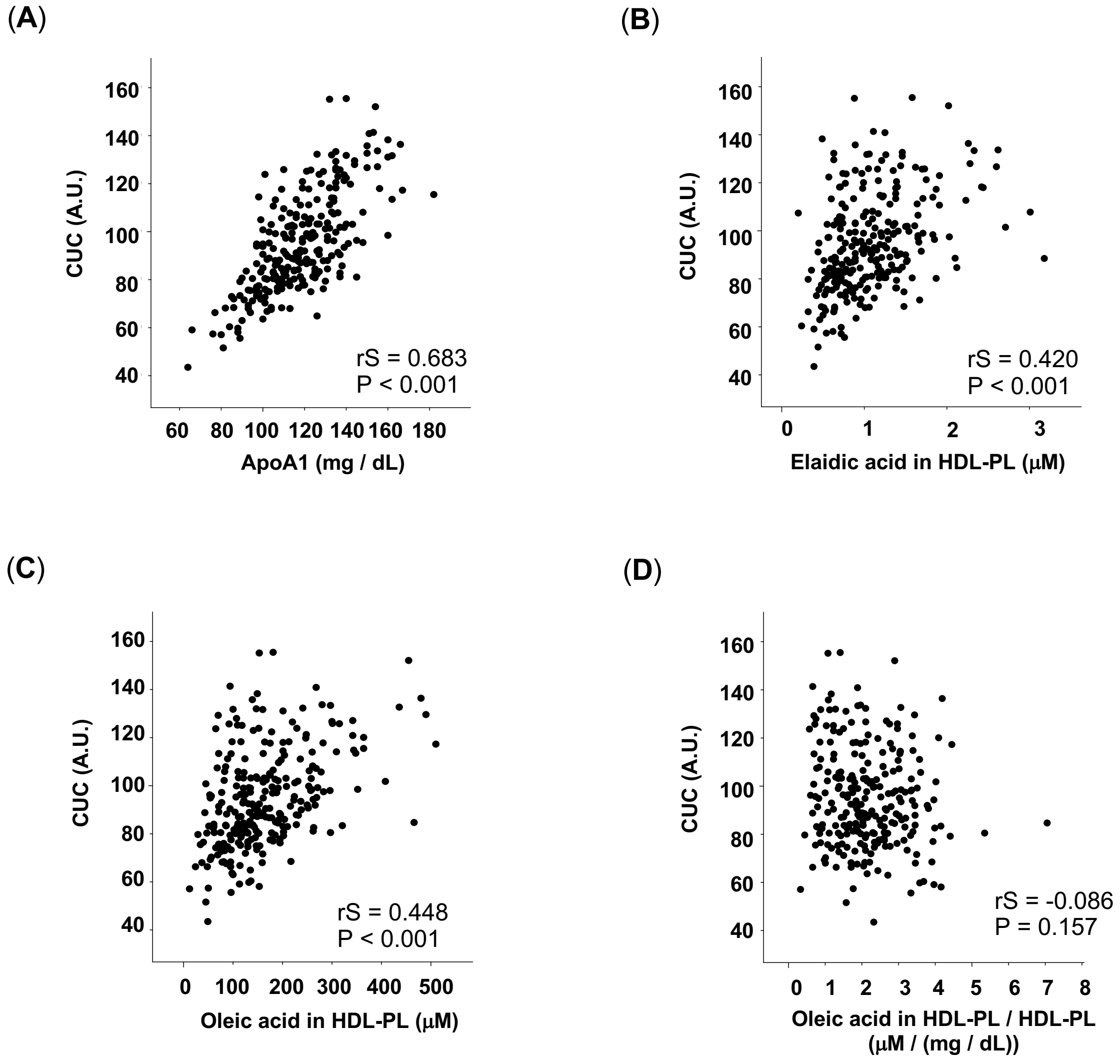

Figure S1. (**A**) Correlations between CUC and apoA1 (rS = 0.683, P < 0.001). (**B**) Correlations between CUC and Elaidic acid in HDL-PL (rS = 0.420, P < 0.001). (**C**) Correlations between CUC and Oleic acid in HDL-PL (rS = 0.448, P < 0.001). (**D**) Correlations between CUC and oleic acid in HDL-PL/ HDL-PL ratio (rS = -0.086, NS). CUC, cholesterol uptake capacity; A.U., arbitrary units; ApoA1,apolipoprotein A1; HDL-PL, high-density lipoprotein phospholipid.
