# Supplementary material for: Effects of Elaidic Acid on HDL Cholesterol Uptake Capacity
Source: Nutrients. 2021 Sep 4;13(9):3112. doi: 10.3390/nu13093112 (PMC8464738; doi:10.3390/nu13093112)
Supplement: Supplementary file 1 [file nutrients-13-03112-s001.zip › nutrients-1336097-supplementary/Supplemetary files (final prrof)/Supplementary_Tables (final proof).pdf]

Table S1. Detailed laboratory data

	Rev.(+) (n=80)	Rev.(–) (n=184)	p Values
**HbA1c (%)**	6.53 ± 1.2	6.3 ± 0.9	0.22
**TG (mg/dL)**	133.9 ± 70.8	126.5 ± 71.3	0.12
**TC (mg/dL)**	146.1 ± 29.9	147.1 ± 31.7	0.74
**LDL-C (mg/dL)**	83.9 ± 26.6	81.2 ± 26.1	0.67
**HDL-C (mg/dL)**	43.2 ± 10.1	47.4 ± 13.4	0.061
**CUC (A.U.)**	90.0 ± 17.8	96.9 ± 21.2	0.026
**HDL-PL (mg/dL)**	71.2 ± 23.1	80.9 ± 27.5	0.015
**HDL-TG (mg/dL)**	13 .1 ± 5.9	13.8 ± 6.5	0.37
**Elaidic acid in HDL-PL (μM)**	0.98 ± 0.43	1.1 ± 0.52	0.065

Values are presented as mean ± SD. Rev.(+), patients with revascularization; Rev.(–), patients without revascularization; HbA1c, hemoglobin A1c; TG, triglyceride; TC, total cholesterol; LDL-C, low-density lipoprotein cholesterol; HDL-C, high-density lipoprotein cholesterol; CUC, cholesterol uptake capacity; HDL-PL, high-density lipoprotein phospholipid; HDL-TG, high-density lipoprotein triglyceride; A.U., arbitrary units. Data were analyzed using the unpaired Mann-Whitney test.

Table S2. Detailed baseline patient characteristics

	Rev.(+) (n=80)	Rev.(–) (n=184)	p Values
Age	71.0 ± 9.9	72.1 ± 8.9	0.39
Male, n (%)	67 (83.8)	143 (77.7)	0.26
Hypertension, n (%)	57 (71.3)	147 (79.9)	0.12

Dyslipidemia, n (%)	64 (80.0)	157 (71.0)	0.28
Diabetes, n (%)	46 (57.5)	73 (39.7)	0.007
Smoking history, n (%)	56 (70.9)	124 (67.4)	0.58

Values are presented as mean ± SD and absolute numbers (%). Rev.(+), patients with revascularization; Rev.(–), patients without revascularization. Data were analyzed using the chi-square test for categorical values and the unpaired Mann-Whitney test for continuous variables.
